# Genomic Analysis of the Emergence and Rapid Global Dissemination of the Clonal Group 258 *Klebsiella pneumoniae* Pandemic

**DOI:** 10.1371/journal.pone.0133727

**Published:** 2015-07-21

**Authors:** Jolene R. Bowers, Brandon Kitchel, Elizabeth M. Driebe, Duncan R. MacCannell, Chandler Roe, Darrin Lemmer, Tom de Man, J. Kamile Rasheed, David M. Engelthaler, Paul Keim, Brandi M. Limbago

**Affiliations:** 1 Translational Genomics Research Institute, Flagstaff, Arizona, United States of America; 2 Division of Healthcare Quality Promotion, Centers for Disease Control and Prevention, Atlanta, Georgia, United States of America; Quuen's University Belfast, UNITED KINGDOM

## Abstract

Multidrug-resistant *Klebsiella pneumoniae* producing the KPC carbapenemase have rapidly spread throughout the world, causing severe healthcare-associated infections with limited antimicrobial treatment options. Dissemination of KPC-producing *K*. *pneumoniae* is largely attributed to expansion of a single dominant strain, ST258. In this study, we explore phylogenetic relationships and evolution within ST258 and its clonal group, CG258, using whole genome sequence analysis of 167 isolates from 20 countries collected over 17 years. Our results show a common ST258 ancestor emerged from its diverse parental clonal group around 1995 and likely acquired *bla*_KPC_ prior to dissemination. Over the past two decades, ST258 has remained highly clonal despite diversity in accessory elements and divergence in the capsule polysaccharide synthesis locus. Apart from the large recombination event that gave rise to ST258, few mutations set it apart from its clonal group. However, one mutation occurs in a global transcription regulator. Characterization of outer membrane protein sequences revealed a profile in ST258 that includes a truncated OmpK35 and modified OmpK37. Our work illuminates potential genomic contributors to the pathogenic success of ST258, helps us better understand the global dissemination of this strain, and identifies genetic markers unique to ST258.

## Introduction

Enterobacteriaceae are a common cause of healthcare-associated bacterial infections, including pneumonia, meningitis, sepsis, and other life threatening illness, especially among patients with underlying medical conditions. The recent rise of carbapenem-resistant Enterobacteriaceae (CRE) has left clinicians with limited antimicrobial treatment options for these infections, and has been declared an immediate public health threat that requires urgent and aggressive action by the Centers for Disease Control and Prevention [1]. *Klebsiella pneumoniae* carbapenemase (KPC)-producing *K*. *pneumoniae* are now one of the most widely disseminated CRE pathogens, and are associated with high morbidity and mortality rates [2, 3]. Since their initial identification in 2001 [4], KPC-producing *K*. *pneumoniae* have emerged throughout the United States (currently identified in 47 states; CDC unpublished data) and the world, spanning five continents that also include South America, Eurasia, Africa and Australia [5–7].

The rapid, widespread dissemination of KPC-producing *K*. *pneumoniae* is largely attributed to the clonal expansion of a single dominant strain, sequence type (ST) 258 as defined by multilocus sequence typing or MLST, currently circulating in over 20 countries [6]. ST258 is a member of the recently designated clonal group (CG) 258 [8], which comprises several other sequence types linked to outbreaks, suggesting that these strains may share genetic features that predispose them to pathogenicity or increased transmissibility. Unlike ST258, other CG258 strains are associated with a variety of carbapenemases including KPC, NDM, VIM, and OXA-48 [9–11]. The transmission of KPC-producing ST258 and other CG258 strains is frequently linked to patient travel or healthcare exposure in known endemic areas, such as the United States, Israel, and Greece [6, 12, 13]. Despite previous genomic analyses of ST258 [14–18], an explanation for its pathogenic success in the healthcare system remains unclear.

Large homologous recombination events frequently shape genomes to result in new emerging pathogens [19]. A sequence of these events has now been documented for CG258 and ST258. Gaiarsa and colleagues, using sequence from Italian isolates and the public database, discovered a putative recombination event that gave rise to CG258. Their evidence shows a donor related to *K*. *pneumoniae* ST1628 contributed ~1.3 Mbp to an ancestor of ST11 (CG258) sometime before 1985 [17]. Chen and colleagues used public genomic data to show the ST258 lineage resulted from a ~1.1 Mbp recombination event between ST11 and a strain related to a Brazilian ST442 isolate [20]. DeLeo and colleagues published a whole genome SNP-based phylogeny of ST258 from mostly the northeastern U.S., and concluded that ST258 comprises two distinct lineages which diverged after a homologous recombination event of ~215kb that included the capsule polysaccharide synthesis (*cps*) locus [15]. Additionally, Wyres and colleagues documented several recombination events involving *cps* loci in CG258 [18].

We contribute to the rapidly growing knowledge base of the KPC-producing *K*. *pneumoniae* pandemic with a geographically, temporally, and genotypically diverse set of isolates, and phylogenetically place ST258 and CG258 within the context of other pathogenic *K*. *pneumoniae*. We propose a timeline for the emergence, *cps* locus divergence, and clinical detection of ST258. We also examine regions of interest of the genomes including mobile elements, single nucleotide polymorphisms (SNPs), *cps* loci, and outer membrane proteins to compare ST258 to the rest of CG258, and to compare CG258 to other strains, to elucidate factors that may contribute to their pathogenic success.

## Results

### Genomics of *K*. *pneumoniae*

**Fig 1:**
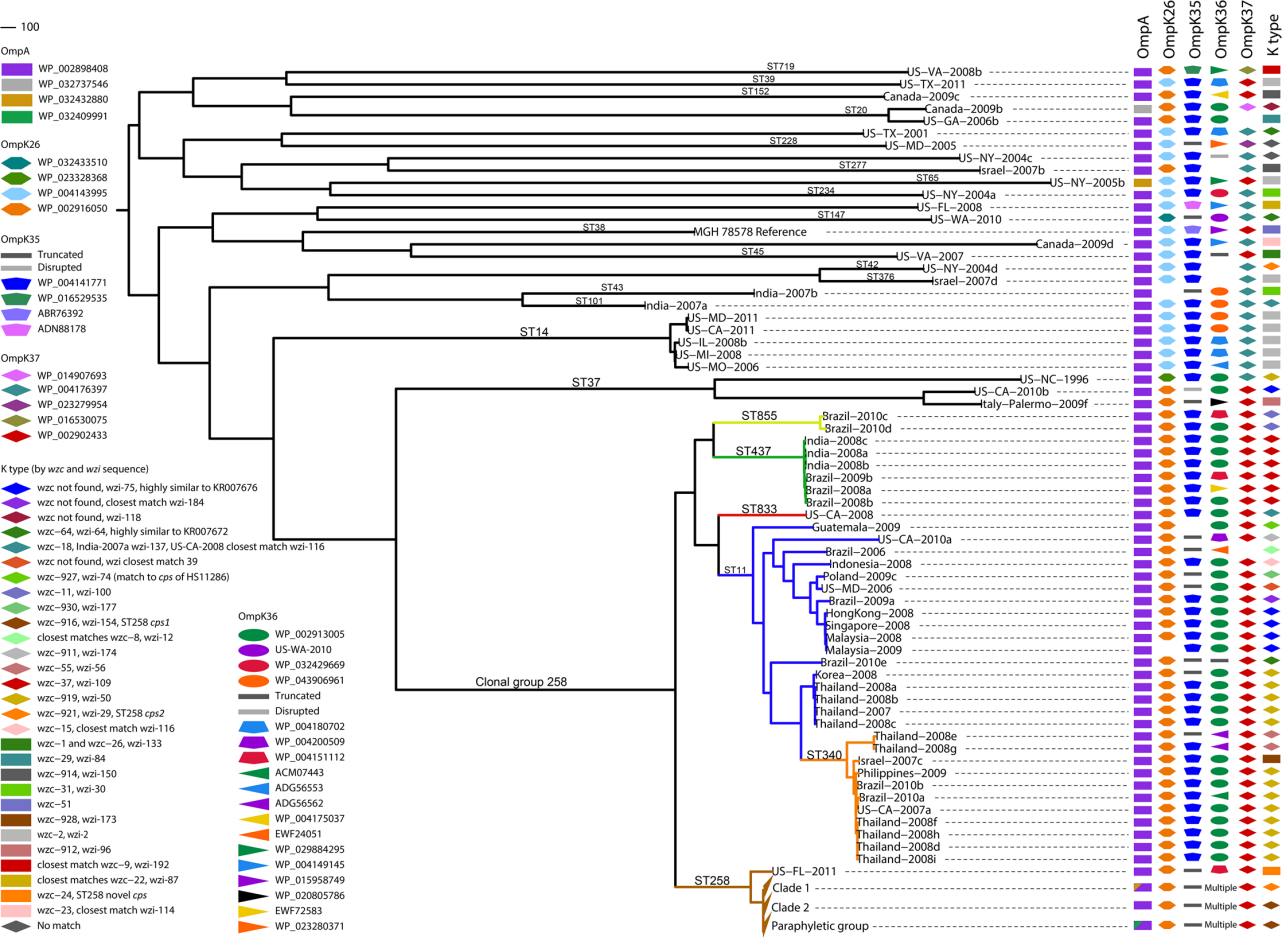
Genetic diversity of healthcare-associated *K*. *pneumoniae*. A maximum parsimony phylogeny based on 49,094 core genome SNPs in 165 *K*. *pneumoniae* isolates and the reference genome MGH 78578 illustrate the diversity of *K*. *pneumoniae* pathogens. The consistency index of the phylogeny is 0.34, reflecting a high number of homoplasious SNPs and indicative of high levels of homologous recombination. (Non-homologous DNA is not analyzed, as it is not part of the core genome.) The main branches of the groups within ST258 are collapsed. In CG258, branches are colored by sequence type. Outer membrane protein sequence was matched by BLAST to a Genbank accession number, except in the case of OmpK36 where matches of high similarity were not always found, in which case the sample name was used as the identifier. The *cps* loci of all strains were characterized by the *wzc* and *wzi* sequences [76, 77] and full-length characterization where genome assemblies allowed.

Our whole genome sequence analyses are based on SNPs, which are inherently stable, that fall in the core genome, or only the regions of the genome homologous to all isolates in the sample set (see details in Materials and Methods). Whole genome analysis of our 167 diverse isolates resulted in a core genome size of 2.2 Mbp. After two clear outliers were removed (a ST334 and a novel sequence type that were more than 37,000 SNPs from their closest neighbor), the core was still small at 2.4 Mbp (Table 1) compared to the known *K*. *pneumoniae* chromosome size of 5.1 to 5.4 Mbp (from publicly available genomes of clinical isolates). This signifies a lack of genomic overlap among the isolates, likely due to a large number of genes acquired through horizontal gene transfer (HGT) and non-homologous recombination. These calculations show, even with a limited number of isolates outside CG258, that *K*. *pneumoniae* is a very diverse species; the average pairwise SNP distance between sequence types is 8,490. The maximum parsimony reconstruction of the phylogeny using the SNP data support this, illustrating diversity even in the homologous regions of the genomes with long branches between isolates (Fig 1)

**Table 1:** Results of mapping Illumina sequencing reads from *K*. *pneumoniae* isolates to relevant reference genomes. For further definition of the calculations see Materials and Methods.

Sample set	No. isolates	Reference genome (genome size)	Total no. SNPs	No. SNPs parsimony informative	Total breadth of reference genome coverage (Core genome size)	Breadth of reference genome coverage all samples	Maximum parsimony consistency index	Illustr.
*K*. *pneumoniae* this study	167	MGH 78578 (5.32 Mbp)	163,711	54,853	40.7% (2.16 Mbp)	>70.8%	0.53	Data not shown
*K*. *pneumoniae* 2 outliers pruned	165	MGH 78578 (5.32 Mbp)	49,094	24,735	44.6% (2.37 Mbp)	>70.8%	0.34	Fig 1
CG258	138	NJST258_1 ~1.1 Mbp masked (4.2 Mbp)	7,256	4,626	50.0% (2.1 Mbp)	>77.6%	0.6	Fig 2A
ST258*	102	NJST258_1 ~215kb masked (5.05 Mbp)	1,425	307	75.5% (3.82 Mbp)	>93.1%	0.96	Figs 2B and3
ST258 from this study* and SRA Study SRP036874**	185	NJST258_1 ~215kb masked (5.05 Mbp)	2,282	582	72.3% (3.65 Mbp)	>93.1%	0.96	S1 Fig

* Including ST512 and ST1199, both point mutations SLVs of ST258

** Including ST379, ST512, and ST418, all SLVs of ST258 [15]

Our analyses provide the resolution to infer evolutionary history and exemplify the limitations of relational inferences from the traditional seven-locus MLST scheme. The adoption of the scgMLST scheme and clonal group nomenclature proposed by Bialek-Davenet *et al*. [8] address these limitations, however a full conversion from MLST-derived “sequence types” has yet to be proposed. Hundreds of SNPs separate ST258 from most MLST single locus variants (SLVs); the average pairwise SNP distance between ST258 isolates and those of the rest of the clonal group is 304 SNPs. Long branches separating isolates of the same sequence type or within the same clonal group, for example in ST37 and within CG258, often signify homologous recombination events like those documented by Gaiarsa, Chen, and DeLeo [15, 17, 20]. In contrast, ST512 and ST1199 are point mutation SLVs of ST258 and are clearly part of the ST258 lineage, and for ease of reading, are referred to as ST258 throughout. ST258 is a closely related group (average pairwise SNP distance of 13) within its diverse ancestral group (average pairwise SNP distance of the remaining isolates in CG258 is 214). This is evidence that ST258 is a recent emergence from the ancestral CG258 clade (Fig 1).

**Fig 2:**
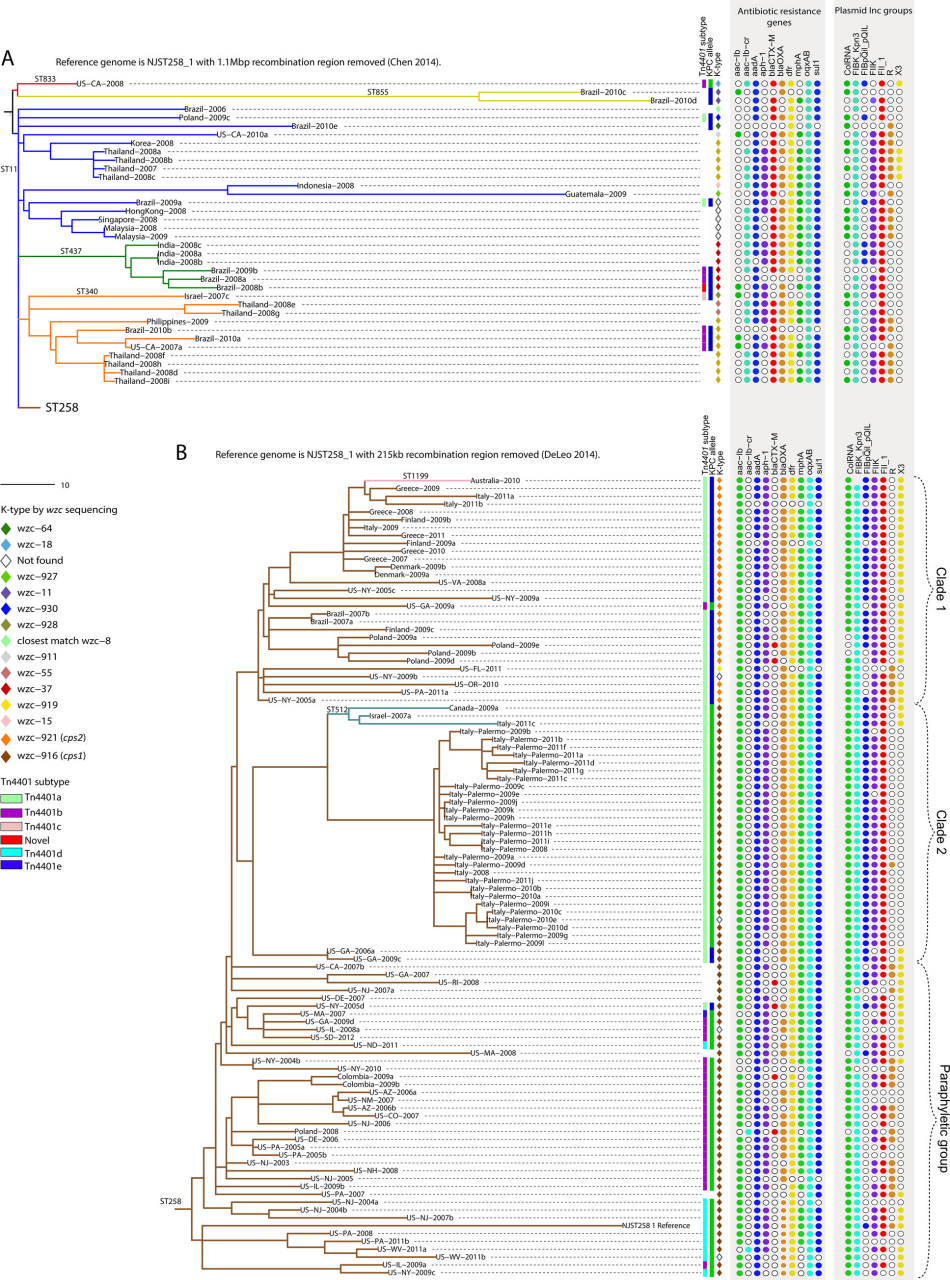
Phylogenies of CG258 and ST258 with large recombined regions removed. (A) A maximum parsimony phylogeny based on 1,440 core genome SNPs in 138 CG258 isolates using NJST258_1 with the 1.06 Mbp region of recombination [20] masked as a reference reduces the genomic distance between ST258 and the rest of CG258. The consistency index of the maximum parsimony phylogeny is 0.95, indicating most SNPs in the core are vertically transferred. (B) A maximum parsimony phylogeny based on 1,425 core genome SNPs in 102 ST258 isolates, using NJST258_1 with the 215 kb region of recombination [15] masked as a reference illustrate the clonal nature and evolutionary history of ST258. The consistency index is 0.96 for the ST258 maximum parsimony phylogeny, indicating most SNPs in the core are vertically transferred. Analysis of antibiotic resistance genes, Tn*4401*, capsule type by *wzc* sequence, and plasmid incompatibility groups lend insight into the vertical versus horizontal transfer of these genetic elements. Complete SRST2 [65] and PlasmidFinder [71] results are in S2 Table.

In order to illustrate the evolutionary history of all members of CG258, we masked the large regions of recombination identified in previous studies from the finished chromosome of the ST258 isolate NJST258_1 [15, 20] to filter non-phylogenetically informative SNP loci. Phylogenies of closely related isolates or defined by few SNPs can be heavily influenced by SNPs in recombinant regions leading to false inferences of evolutionary history. Reads from the 137 isolates in CG258 were mapped to NJST258_1 and the 1.06 Mbp recombinant region [20] was masked, resulting in a reference genome of 4.2 Mbp. A significant reduction in the pairwise SNP distance comparison between ST258 and the rest of CG258 resulted; an average of 62 SNPs separate the isolates from the two groups (compared to 304 stated above). The phylogeny from these data illustrates that ST11 is a paraphyletic group with respect to ST437, ST340, and ST258, and only four SNPs distinguish the ST258 lineage from its clonal group (Fig 2A). The core genome in this analysis is 50% of the 4.2 Mbp reference genome, or 2.1 Mbp (Table 1), implying that there are many regions of non-homologous recombination in this sample set.

**Fig 3:**
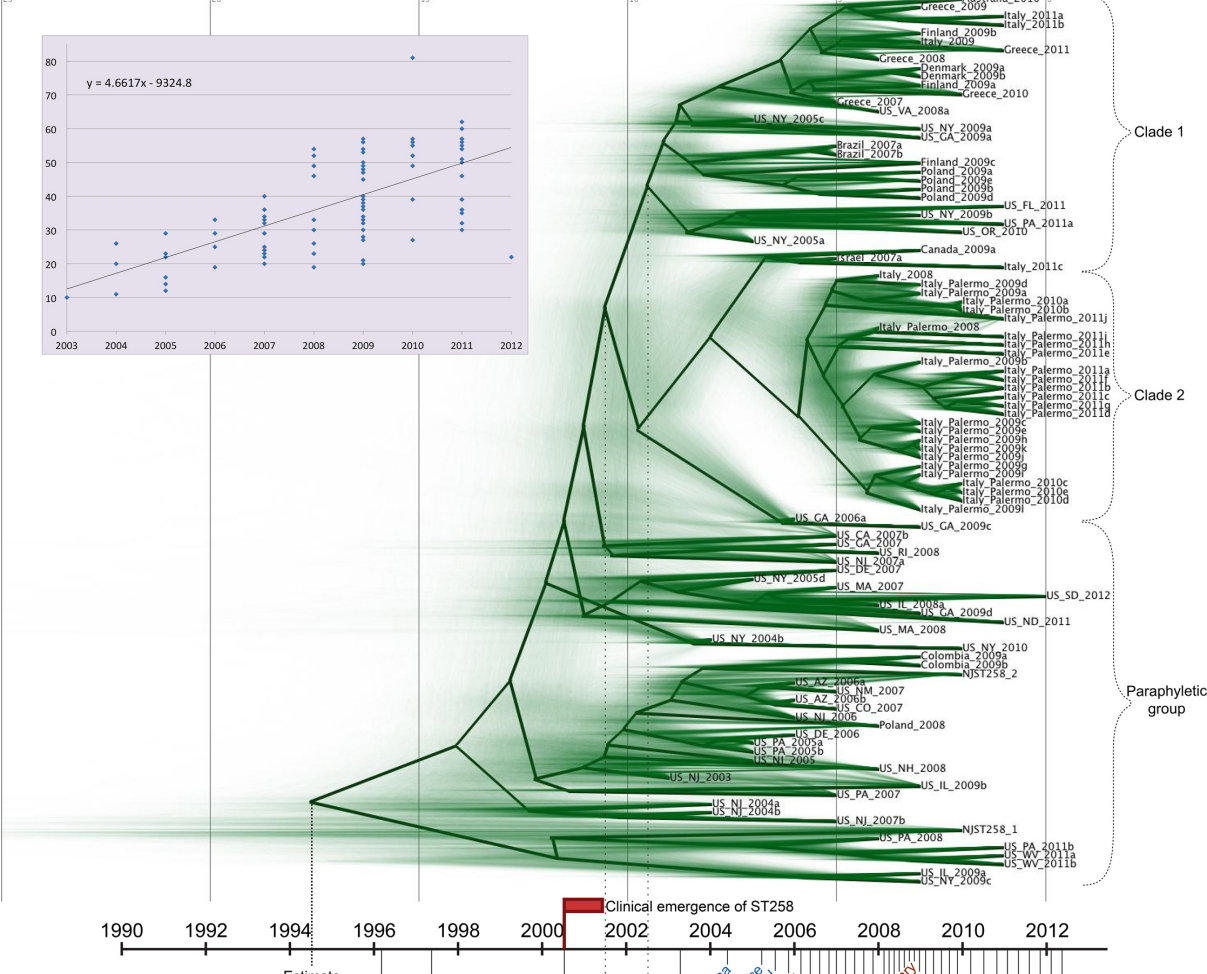
Projecting the evolutionary history of ST258. BEAST analysis based on 1,425 core genome SNPs in 101 ST258 isolates with NJST258_1 reference genome, with the 215 kb region of recombination [15] masked, gives temporal context to the emergence of ST258, with key events and initial reports of KPC-producing *K*. *pneumoniae* in different countries plotted. Blue font indicates reports of KPC-producing *K*. *pneumoniae*, brown font is ST258. Green shading on the phylogeny shows lines of iterations of Bayesian analyses. The mean mutation rate of *K*. *pneumoniae* ST258 is 1.03 x 10^−6^ (95% HPD 8.09 x 10^−7^ to 1.24 x 10^−6^). The TMRCA for the ST258 clade is approximately 20 years ago, around 1995. The plot inset is a root-to-tip analysis of SNP accumulations for each isolate since the MRCA of ST258. The slope of the fit line is 4.66, which is close to the mutation rate calculated by BEAST ((1.03 x 10^−6^ substitutions per site per year) x (3.8 Mbp core genome size) = 3.9 SNPs per year).

When reads from the 101 isolates in the ST258 group were mapped to the complete NJST258_1 (Table 1) chromosome, the ~215kb region of homologous recombination [15] was identified by its high SNP density; 971 out of the total 2,396 SNPs fell in this region. This region was masked from the NJST258_1 reference to generate a refined ST258 SNP matrix. Compared to the entire collection, the core genome of ST258 is considerably larger at 3.8 Mbp (Table 1) due to more genome content in common, emphasizing the clonality of this group. The refined SNP matrix was used in both maximum parsimony and Bayesian (BEAST) analyses (Figs 2B and 3). Resulting phylogenies showed comparable overall topologies with the monophyletic Clades 1 and 2 originally defined by DeLeo *et al*. [15] conserved, and also sharing a common ancestor of their own. The remaining isolates are paraphyletic with respect to Clades 1 and 2. Within the context of ST258’s genetic relationships with other *K*. *pneumoniae*, our data illustrate that ST258 isolates are of a single clonal lineage derived from a recent common ancestor.

### Emergence and evolution of ST258 with KPC

Bayesian analysis estimates the time to most recent common ancestor (TMRCA) of the global ST258 group as 17.2 years before our most recent isolate collected in 2012, or around 1995 (95% highest posterior density [HPD] 12.3 to 23.1 years, Fig 3), slightly different from the conclusions of Gaiarsa *et al*. [17], who calculated the year 1997. From this study, US-NJ-2003 is the earliest confirmed ST258 to date. Previous reports have linked ST258 to a KPC-producing hospital outbreak in New York City in April, 2000 [21], suggesting that ST258 emerged as clinically significant just 5 years after origination. Notably, the first KPC-producing isolate identified was a ST37 strain collected in 1996 in North Carolina [4], contemporaneous and proximal with our estimates of the first ST258 strains. The estimated time of the recombination event resulting in one of the alternate *cps* loci [15] is around 2001 to 2003. We observed a strong correlation in the KPC-producing ST258 between *cps1*-containing isolates with KPC-2 (95%), and *cps2*-containing isolates (all in Clade 1) with KPC-3 (97%, Fig 2), suggesting the KPC gene point mutation occurred in the common ancestor to Clade 1 between 2001 and 2003 as well. KPC-3-producing *K*. *pneumoniae* were first collected around this time [22], likely from ST258 strains [21], supporting this idea.

To enhance our collection of ST258 genomes, published sequence reads from 83 ST258 isolates mostly collected from the northeastern U.S. (NCBI SRA database study SRP036874, [15]) were added to the analysis with the masked NJST258_1 reference genome. A total of 2,282 SNPs were identified among all 186 ST258 isolates and were used in a maximum parsimony analysis and a second BEAST analysis that estimates TMRCA at 16.4 years before the 2012 isolate (95% HPD 12.8 to 20.6 years), both of which corroborate our previous estimates. We also incorporated the sequence data of 22 isolates from the NIH outbreak in 2011 [23] to illustrate the phylogeny of all 208 isolates (S1 Fig). The publicly available genomes fall interspersed with ours throughout the phylogeny, showing that isolates from the northeastern U.S. and Canada genetically reflect a global isolate collection, supporting a northeast U.S. origination or highlighting the region as a hub of global travel. Clade 1 includes 29 publicly available genomes and remains monophyletic with four SNPs common to all. Clade 2 no longer has SNPs in common with the Georgia isolates on a basal branch in Fig 2 (Clade 2 bottom branch, and boxed in S1 Fig), and these isolates were the only KPC-2 producers that fell in Clade 2. The NIH outbreak isolates clustered in their own tight clade, but monophyletic, having five SNPs in common, with the clade of Palermo, Italy, outbreak isolates (S1 Fig).

Our SNP phylogenies corroborate transmission of strains previously suggested to have epidemiologic linkages and highlight previously unrecognized transmission events. In addition, by juxtaposing variable genetic features alongside the SNP-based phylogeny, we obtain further insight into epidemiologic and genetic transmission events. For example, 13 isolates, most collected from patients with a recent history of travel or healthcare exposure in Greece, clustered together despite being collected from diverse locations including Australia, Denmark, Finland, Greece, and Italy (Fig 2, Clade 1) [24–26]. The small distance (average pairwise distance 21 SNPs) found among these isolates and shared genetic features indicate a common source. Also, an isolate collected in 2008 from Florence, Italy, unexpectedly clusters tightly with 27 isolates from a multi-institutional ST258 outbreak in Palermo, Italy. This isolate, Italy-2008, was the first ST258 identified in Italy, and was previously linked to Israel [27].

### Mobile elements

The KPC gene, *bla*_KPC_, is carried on a highly mobile transposable element, Tn*4401* [28] that is passed both vertically through bacterial clonal expansion and horizontally between unrelated strains. Tn*4401* subtypes have different deletions upstream of *bla*_KPC_ that confer different promoter regions to the gene [29]. Tn*4401b* is the full-length Tn*4401* element; different deletion events result in conversion to *a*, *c*, *d*, and *e* subtypes. Sequential deletions could be responsible for subtype conversions; the most plausible based on deletion size are conversions from *a* or *d* to *c* or *e*, and *c* to *e*. However, selection may be against conversions from Tn*4401a* or *d* as strongest *bla*_KPC_ expression occurs with the promoters present in these subtypes [29]. Vertical transfer of *bla*_KPC_ within a limited period of time and on a local or regional level has been observed previously [30, 31]. Parsimony analysis of our data suggests that the vertical transfer of Tn*4401* in ST258 has played a significant role in *bla*_KPC_ dissemination (Fig 2B). The paraphyly that characterizes isolates outside of Clades 1 and 2 suggests that the ST258 MRCA carried the full length Tn*4401b* with *bla*_KPC-3_ and that deletion events in Tn*4401* and point mutations in *bla*_KPC_ occurred before various clades diverged. Occasionally, independent acquisition by horizontal transfer is evident where an isolate carries Tn*4401b* while the majority in its clade have another subtype, as is the case for US-GA-2009a (Fig 2B, Clade 1), which also differs in *bla*_KPC_ type from its closest relatives, and possibly for US-IL-2009a (Fig 2B bottom, cluster containing Tn*4401d*). US-NY-2005a carries both a Tn*4401a* and Tn*4401b*. This isolate may still be carrying the ancestral *b* and acquired *a*, or may have acquired *b* in addition to its *a*, or this may be the result of a transposon duplication event. Independent Tn*4401* acquisition in ST258 is indicative of persistent selective pressure for this dominant strain to harbor *bla*_KPC_ or genes that may be acquired along with it (*i*.*e*. other antibiotic resistance genes carried on the same mobile element).

Conservation of plasmid incompatibility groups within ST258 may reflect the successful vertical transmission of particular plasmids. The predominant incompatibility groups in ST258 are FIB_K_ (pKPN3, in 96% of isolates), ColRNA (in 95%), FII (in 86%), and FII_K_ (in 82%). The KPC-encoding plasmid pKpQIL described in ST258 outbreaks in Israel and Italy [32, 33] and pKpQIL-like plasmids in New Jersey and New York isolates [34] are multi-replicon plasmids of both incompatibility groups FII_K_ and FIB (pKpQIL). Within Clades 1 and 2, nine isolates do not have these plasmid types, one of which is US-GA-2009a that appears to have lost and reacquired Tn*4401* (Fig 2B). Our phylogeny suggests that the MRCA to Clades 1 and 2 harbored a pKpQIL-like plasmid that was then lost as few as five times. The FIB type of pKpQIL plasmids is absent from most isolates outside of these clades. The plasmid type profiles of Clades 1 and 2 are very similar overall; most isolates have five of the seven types illustrated in Fig 2B. IncX appears to have been lost in the Italy-Palermo isolates (and also appears to have been lost in another clade in the paraphyletic group). Incompatibility types are diverse in the paraphyletic group of isolates. Plasmids, therefore, likely add considerably to the *K*. *pneumoniae* species pan-genome. The seven *bla*_KPC_-negative ST258 isolates may have lost a KPC-encoding plasmid, as has occurred before [35], however, no clear incompatibility group pattern correlates strongly with its loss. Three of them appear to carry a pKpQIL-like plasmid. Likewise, outside of ST258 in CG258, three Tn*4401*-negative isolates in ST437 also appear to carry a pKpQIL-like plasmid. And again, no clear incompatibility group pattern correlates strongly with Tn*4401* carriage. These observations are not surprising as Tn*4401* is associated with many different plasmid types owing to its high mobility [36–39].

An abundance of resistance genes are harbored by CG258, presumably on various plasmids (though indeterminate from these data, Fig 2). These genes encode mechanisms of resistance to quinolones (*aac-Ib-cr* or *oqxAB* identified in 100% of isolates), aminoglycosides (*aac-Ib*, *aadA*, or *aph-1*, in 99% of isolates), β-lactams (by β-lactamases encoded by *bla*_KPC_, *bla*_CTX-M_ or *bla*_OXA_, in 96%), trimethoprim and sulfonamides (*dfr* or *sul1* in 93% and 94%), and macrolides (*mphA*, in 81%), many of which were also identified, although less frequently, in unrelated *K*. *pneumoniae* (S2 Table). The antibiotic resistance profile these genes confer is highly similar in ST258 and the rest of CG258. At the gene level, ST258 differs from the rest of CG258 in *bla*_KPC_ and *bla*_CTX-M_ status, and in *aac-Ib* and *aac-Ib-cr* status. The high frequency of *bla*_CTX-M_ in CG258-non-ST258 may be due to sampling bias, however these sequence types are known to often carry β-lactamase genes [9–11]. Interestingly, the quinolone resistance gene *aac-Ib-cr*, in many of the CG258-non-ST258 isolates but in only two ST258 isolates, is two nucleotides different from the aminoglycoside resistance gene *aac-Ib*, present in almost all ST258 and in only six of the rest of CG258 isolates. Both of the *aac-Ib-cr*-positive ST258s are *aac-Ib*-negative, and vice versa for the *aac-Ib*-positive CG258-non-ST258s, suggesting that these two genes are not independently acquired in the two groups, but the MRCA to all carried one and point mutations result in the other. If this is the case, the point mutations have happened in more than one lineage in both groups.

The frequent point mutations observed in CG258 in *aac-Ib* and *aac-Ib-cr* are interesting in that all CG258 isolates have the fluoroquinolone resistance-conferring mutations in GyrA (Ser83 to Ile) and ParC (Ser80 to Ile), the DNA gyrase and topoisomerase enzymes on which fluoroquinolones act, and almost all have another aminoglycoside resistance gene, *aadA* or *aph-1*. This resistance is important considering fluoroquinolones and aminoglycosides are drugs of choice for urinary tract infections (UTIs), the major pathology caused by ST258. Also, all CG258 isolates carry the genes for the OqxAB efflux pumps, generally conferring low-level resistance to fluoroquinolones. The apparent selection pressure for multiple mechanisms of similar resistance may be due to the slightly different phenotypes conferred by each.

We screened our isolate genome assemblies for several virulence genes recently described in the highly virulent capsule type K2 Kp52.145 isolate [40] to determine their potential contribution to pathogenic success. Within CG258, we found several instances of colibactin genes, which encode a genotoxin that induces host DNA damage, and conjugation machinery of type IV secretion systems (T4SS), which is not surprising given CG258’s plethora of plasmids. Of note, we found two isolates that carry genes similar to the newly described *pld1* gene encoding a phospholipase D protein involved in lipid metabolism [40]. PLD1 was found to be prevalent in highly virulent *K*. *pneumonia*e isolates or those known to cause severe infections [40]. In our collection, ST39 (US-TX-2011) and ST719 (US-VA-2008b), but no CG258, carried genes similar to *pld1*.

### Factors impacting extracellular interaction

Among the four SNPs that separate all ST258 from the rest of CG258, one is non-synonymous in a gene encoding a transcriptional regulator protein in the multiple antibiotic resistance repressor (MarR) family. Members of this family such as SlyA in Salmonella and MarR in *E*. *coli* and *K*. *pneumoniae* (different from this MarR family protein) have a helix-turn-helix motif and form homodimers that bind DNA at marboxes to block expression of genes (S2 Fig). MarR family proteins also bind stimulatory ligands thought to result in a conformational change averting its bond with DNA. In this way, MarR family proteins mediate metabolic responses to a cell’s environment. Mar regulons have many regulatory functions in many taxa, including multidrug efflux pump and outer membrane porin production, stress tolerance, toxin degradation, and many other virulence factors [41]. MarR is a repressor of the pleiotropic *marRAB* regulon. MarA is a gene expression activator that in *E*. *coli* is involved in regulation of over 60 genes [42, 43], and binds intrinsic copper released upon disruption of cellular membrane processes [44]. MarA is closely associated with and interacts with several other pleiotropic transcription regulators, including SoxS, Rob, and RamA, which all contribute to regulation of the AcrAB-TolC efflux pump genes implicated in fluoroquinolone and tigecycline resistance [42, 45–47]. Overexpression of RamA results in lipopolysaccharide modifications that alter the outer layer of the cell, decreasing its susceptibility to host-derived antimicrobial peptides as well as polymyxins, and increasing its evasion of phagocytosis by host macrophages [46]. The SNP in the *marR*-family gene in ST258 isolates, which appears to be 100% specific and 100% sensitive to ST258 by *in silico* validation, confers Ser34 to Phe amino acid change in the homodimerization region of the protein (S2 Fig). This substitution may affect the proteins’ ability to form the homodimer, which in turn would affect its ability to bind ligands or marboxes. It is conceivable that, considering the potential interconnection of this regulator with others, this amino acid substitution may result in significant metabolic changes in ST258. Indeed, this MarR family protein is very highly conserved among *K*. *pneumoniae*, signifying its functional importance. The only other amino acid changes we found in the species are in KP5-1 and 342, both plant-associated isolates, and interestingly, in all of CG258. The Arg4 to Ser mutation in CG258 occurs in a seemingly insignificant domain of the MarR family protein (S2 Fig); however this change may affect protein folding and therefore function. Additionally, isolates in CG258 share a synonymous nucleotide substitution in the gene (another rare occurrence), C408 to T, which appears to be 100% specific and 100% sensitive to CG258 by *in silico* validation. Although it appears that CG258 inherited this gene from a relative of ST1628 in the 1.3 Mbp recombination event [17], the ST1628 isolate does not share this *marR*-family gene SNP or the Arg4 to Ser amino acid change with CG258.

We capitalized on the specificity of these SNPs in the *marR*-family gene by developing assays to target them (Table 2). These assays could be used in real-time PCR or in amplicon sequencing to detect a CG258 and/or ST258 strain. We screened them as dual-probe real-time PCR assays across a subset of our collection, and found them to be 100% specific and 100% sensitive, correctly typing 48 CG258 and 24 ST258. The ST258 assay was also robust to *K*. *pneumoniae*, correctly typing 49 non-ST258 isolates comprising more than 20 different sequence types. The CG258 assay detected 13/15 non-CG258 isolates. The two it missed are the divergent US-PA-2001 and US-GA-2009b; these two isolates contain SNPs in the *marR*-family gene in the assay region.

**Table 2:** SNP mutations and real-time PCR assays to detect CG258 and ST258. Lowercase letters in the probes indicate the targeted SNP state.

Gene	SNP	SNP Specificity	Assay	Primer/Probe Sequence	Assay Sensitivity/ Specificity
*marR-*family	C101 to T	ST258	ST258_F	ATGGTGGTGCGCCAGTG	To ST258: 100%/100%
			ST258_R	GCTGACCGAGACGTTGTC	To non-ST258: 100%/100%
			ST258-T_FAMBHQ+	CATTATTGACTtCGCTATCA	
			nonST258-C-TETBHQ+	CCATTATTGACTcCGCTAT	
*marR-*family	C408 to T	CG258	CG258_F	ACGGCAGGCGATTTGATTTAACG	To CG258: 100%/100%
			CG258_R	AGCTGCGTGATCGAGACCTATC	To non-CG258: 87%/100%
			CG258-A_FAMBHQ+	CGCTGAAGGTaGCGAGAT	
			nonCG258-G_TETBHQ+	CTGAAGGTgGCGAGATC	
*marR-*family	A12 to C	CG258	Not designed		
*oqxR*	T389 to C	Most CG258	Not designed		

A second transcription regulator that potentially shapes the CG258 phenotype is the repressor OqxR of the OqxAB efflux pump genes. The *oqxAB* locus, originally described on a plasmid in *E*. *coli* [48], is widely reported in *K*. *pneumoniae* [49]. Bialek *et al*. recently described a mutation in OqxR that results in overexpression of OqxAB, which contributed to antibiotic resistance in *K*. *pneumoniae* clinical isolates, and showed that various classes of antibiotics (fluoroquinolones, chloramphenicol, and β-lactams) are among the OqxAB pump substrates [50]. Zhong *et al*. also associate OqxAB with tigecycline resistance [47]. We found a mutation in OqxR in CG258, Val130 to Ala, due to SNP T389 to C that appears to be specific to our CG258. (ST11 HS11286, Genbank accession CP003200, does not have the *oqxAB* locus, however, so it does not appear to be 100% sensitive to CG258.) Veleba *et al*. found Val130 to Ala, but did not associate this mutation with increased or decreased repression citing confounding effects of other metabolic regulators; however, they mention this mutation is part of current experiments [51]. Italy-Palermo-2009g (ST258) also has a deletion of 13 bases that results in a premature stop codon, likely resulting in a defective repressor protein. Three other isolates outside CG258 also have deletions resulting in truncated proteins (two ST14 isolates and US-GA-2009b of novel sequence type), indicating that mutation of this repressor may be a common mechanism of increased antibiotic resistance in clinical isolates.

We characterized the *cps* locus and outer membrane protein (OMP) profiles of our collection, two complex systems that encode multiple proteins in direct contact with the extracellular environment and potential antigenic targets for the host immune system. *K*. *pneumoniae* capsules play an important role in virulence, and CPS modification has been described in other species to allow evasion of host immune detection [52, 53]. We found a remarkable degree of *cps* diversity in our collection, with over 35 different variants falling into over 25 different K-types (Fig 1). CG258 alone contains 18 different variants, of which only seven have been characterized [15, 18], and several have no exact match in the *wzc* and *wzi* gene sequence databases. Recombination in the *cps* region is apparent throughout the phylogeny where K-types (by *wzc* and *wzi* sequence) match between distantly related isolates (Fig 1). In one case, three distantly related isolates, Brazil-2010e (ST11), US-WA-2010 (ST147), and US-TX-2001 (novel ST), have the same full length *cps* sequence highly similar to Genbank accession number KR007672 from another ST11 isolate [18]. Interestingly, characterization of the full *cps* loci showed that all three of our ST37 isolates are characterized by different *cps* loci, and each is shared with CG258 strains; two are shared among isolates in this study (Fig 1), and one *cps* locus was characterized in a ST11 isolate previously [18]. In more than one case, we observed K-type matches by *wzc* or *wzi* sequence, but sequence divergence in other regions of the *cps* locus. Two distantly related isolates share a *wzc* sequence but not *wzi* (Fig 1), and in two cases *wzc* and *wzi* sequences matched between isolates but the full locus did not match.

**Fig 4:**
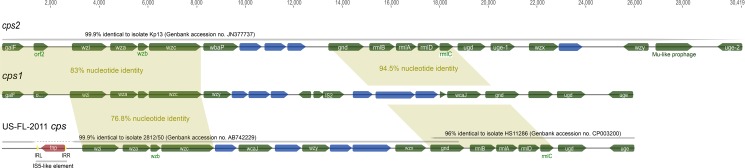
Characterization of three *cps* loci found in ST258 isolates. Regions of identity are shaded and GenBank BLAST matches labeled. Putative glycosyltransferases are in green and hypothetical proteins are in blue. The IS5-like element disrupting the 5’ region of the US-FL-2011 *cps* locus is in red and yellow.

The majority of our ST258 maintained either *cps1* or *cps2*. Our genomic data second the suggestion by DeLeo and colleagues [15] that a ST258 lineage recombined with DNA from a ST42 strain and acquired the *cps1* locus primarily found in ST258 Clade 1. The ST42 isolate in our collection was collected from a Brooklyn hospital in 2004, and the ST42 isolates described in the DeLeo study were collected from New York City hospitals in 2001 to 2002. Our Bayesian analysis calls the *cps1* clade monophyletic; the recombination event that introduced *cps1* in ST258 likely occurred once in a common ancestor to the clade around 2002 (Fig 3), possibly in the New York City area. However, our observation of the strong correlation between *cps1*-containing ST258 with KPC-2, and *cps2*-containing ST258 with KPC-3 (Fig 2), taken in the context of the entire ST258 phylogeny, leads us to hypothesize the *bla*_KPC_ point mutation occurred around the time of the *cps* recombination, rather than from independent acquisition of Tn*4401*, as DeLeo and colleagues suggest [15]. Within Clade 1, our analysis also identified a third ST258 *cps* locus in US-FL-2011. This locus is identical to part of the capsule type K23 isolate, 2812/50 (GenBank accession no. AB742229), but is disrupted by an IS5-like element in its 5’ end, is missing *gal*F and *orf*2, and in part resembles HS11286 (Fig 4). US-FL-2011 was collected as part of a hospital outbreak investigation, suggesting these additional *cps* modifications do not impact ST258 success.

*K*. *pneumoniae* outer membrane proteins not only provide structure to the membrane and allow transport of iron, nutrients, and antimicrobial agents via their pores, but also contain extracellular loops that affect surface adhesion and invasion, biofilm formation, and host immune detection [54–56]. We examined sequence of the major porin proteins KpOmpA, OmpK26, OmpK35, OmpK36, and OmpK37 to explore differences in our collection (Fig 1). KpOmpA demonstrated little variation regardless of sequence type; 95% matched GenBank accession WP_002898408. Only five other variants comprised the rest. KpOmpA interacts with plasmid conjugation machinery; its presence increases frequency of conjugation [56]. KpOmpA can also form two different conformations, resulting in two different membrane pore sizes, offering a form of variation in the protein [56]. OmpK26 was also conserved among isolates; 86% matched GenBank accession WP_002916050, and only five other variants comprised the rest. OmpK26 is indispensable to a cell when OmpK35 and OmpK36 proteins are deficient [57].

**Fig 5:**
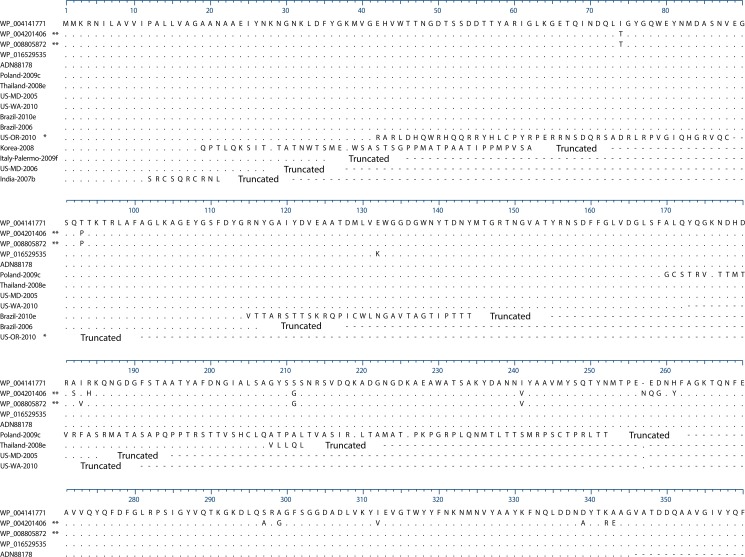
OmpK35 alignment of all alleles found in the 167 isolates. Sequences are labeled by Genbank accession number when they’re an exact match. WP_004141771 was the most frequently found complete protein in our isolates, and was used as the reference in the alignment. Dots are conserved sites, dashes are sites downstream of a premature stop codon. * US-OR-2010 represents all ST258 isolates in the study. ** These variants are in the divergent isolates US-PA-2001 and US-GA-2009b, not shown in Fig 1.

**Fig 6:**
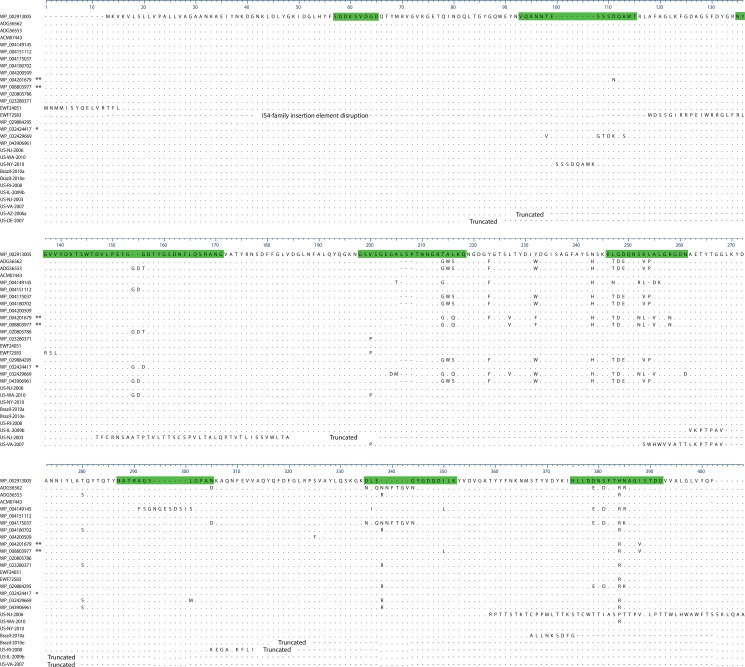
OmpK36 alignment of all alleles found in the 167 isolates. 100% identity BLAST matches were not found for several sequences; sample names are used for these sequences. WP_002913005 was the most frequently found protein so was used as the reference. Sequence in green represents the extracellular loop regions of the protein. Dots are conserved sites, dashes are gaps or represent sites downstream of a premature stop codon. * This variant is not shown in Fig 1; it occurs in ST258 isolate US-GA-2007. ** These variants are in the divergent isolates US-PA-2001 and US-GA-2009b, not shown in Fig 1.

**Fig 7:**
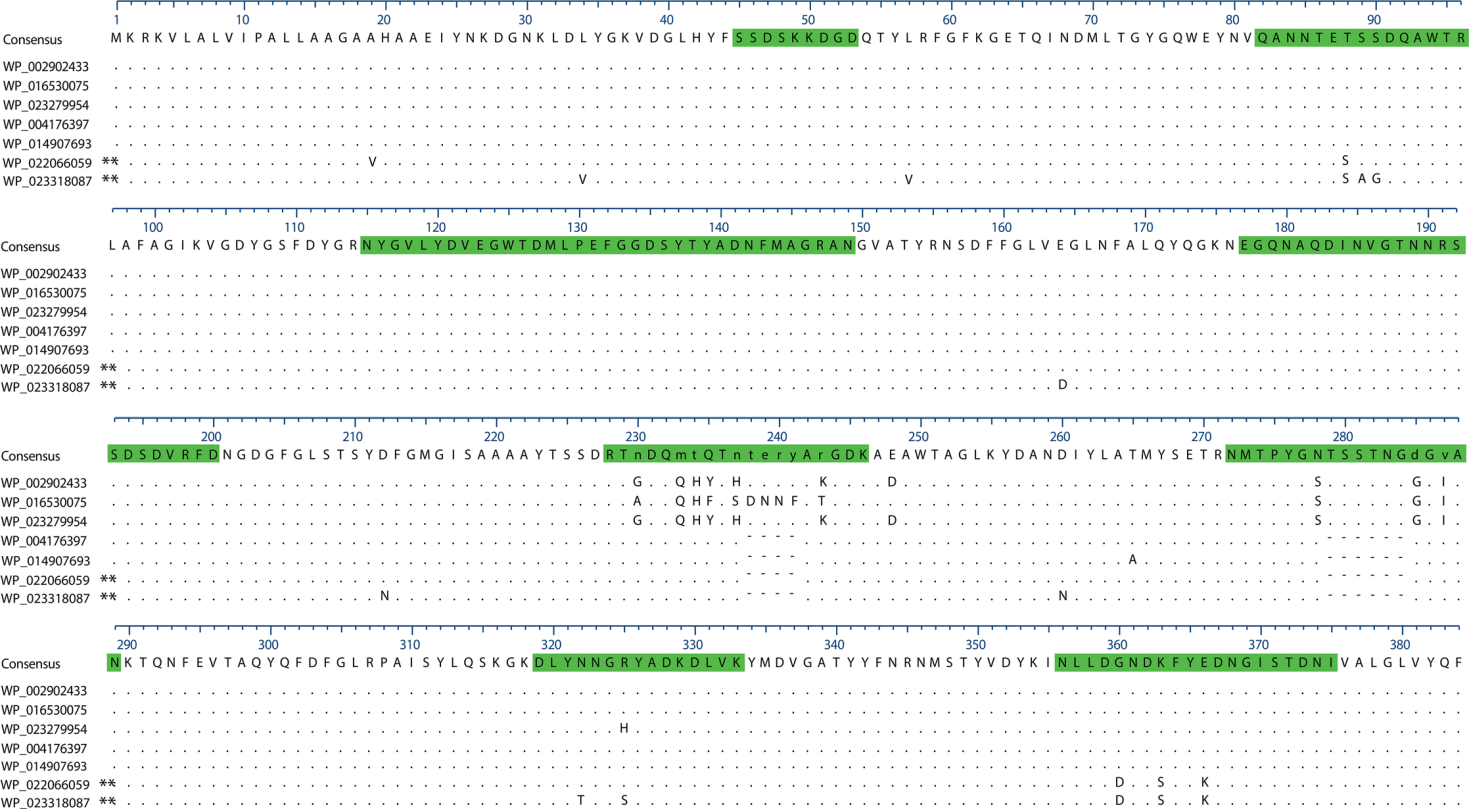
OmpK37 alignment of all alleles found in the 167 isolates. Sequence in green represents the extracellular loop regions of the protein assumed from the structure of OmpF by Doménech-Sánchez *et al*. [59]. Dots are conserved sites, dashes are gaps. ** These variants are in the divergent isolates US-PA-2001 and US-GA-2009b, not shown in Fig 1.

All ST258 isolates in our collection shared an OmpK35 sequence containing a frame-shift that results in a premature stop codon and truncated protein. Although this mutation has been reported previously [58], we found it exclusive to our ST258 group (Fig 1). The resulting outer membrane porin loss increases β-lactam resistance [59] and in combination with a β-lactamase results in high levels of β-lactam resistance [60]. Several other isolates harbored truncated OmpK35 proteins (Figs 1 and 5), likely owing to OmpK35’s allowance of carbapenem antibiotics across the cell membrane, and all harbored a β-lactamase gene, *bla*_KPC_ (n = 5 outside ST258), *bla*_CTX-M_ (n = 4), or *bla*_VIM_ (n = 1). OmpK36 displayed the most diversity (Fig 1, S2 Table); 40 different variants were found, with amino acid variations concentrated in the extracellular loop regions of the protein (Fig 6), presumably diversifying *K*. *pneumoniae*’s interactions with the environment and potentially influencing host immune response and adherence of the cells to host surfaces [55]. Most CG258 shared a similar OmpK36 matching GenBank accession WP_002913005 (76% of ST258 and 76% of CG258), with the remainder sharing 12 variants (Fig 6). In the 155 isolates for which a complete OmpK36 protein was characterized, the average pairwise distance is 3 amino acids. Each unique variant differs by an average of 13 amino acids. Seven isolates have a premature stop codon and putatively non-functioning protein, five of which are ST258. In one isolate, an IS4-family insertion disrupts the 5’ end of the gene. These five mutations do not appear to have clonally spread, as each is unique and these isolates do not fall in the same clades. This may reflect selection against OmpK36 truncation; indeed previous reports associate OmpK36 loss with increased susceptibility to phagocytosis [55]. The OmpK37 sequences of 100% of CG258 isolates match Genbank accession WP_002902433, whose amino acid sequence shows extensions in extracellular loop regions (Fig 7). Outside CG258, 26% of isolates carried this protein, and 66% match Genbank accession WP_004176397 or WP_014907693, which have shorter extracellular loops in regions L5 and L6. The two divergent isolates not shown in Fig 1 both had unique protein sequences for all five proteins. The combination of the extended loop regions in OmpK37 and the absence of a functional OmpK35 are not unique to the ST258 group in our study, however only nine isolates outside ST258 have this profile. The combination of this profile with other characteristic features in ST258 likely impacts extracellular interaction with the environment.

## Discussion

Perfect examples of large homologous recombination events between unrelated strains resulting in new, more successful pathogens [61] are the evolutionary events that generated CG258 [17] and the ST258 strains [20], both results of single events that encompassed approximately 20% of the *K*. *pneumoniae* genome. Whether these events are the secrets to CG258 and ST258’s success is unclear. Over the past decade, CG258 strains carrying carbapenemase genes, especially KPC-producing *K*. *pneumoniae* ST258, have become some of the most successful multidrug-resistant bacterial pathogens in healthcare settings throughout the world. Reports describe ST258’s ability to overtake a previously established carbapenemase-producing *K*. *pneumoniae* strain within an institution in Greece [62], and to rapidly disseminate throughout a country and surpass a pre-existing KPC-producing *K*. *pneumoniae* population in Israel [13, 21]. As other KPC-producing *K*. *pneumoniae* preceded ST258, this suggests that KPC alone is not the driver of ST258’s success, and that ST258 has other evolutionary advantages.

The combination of our parsimony analysis and the coincident emergence of ST258 and KPC around 1995–1996 leads us to propose that ST258’s common ancestor acquired KPC-encoding Tn*4401* prior to dissemination. KPC-producing ST258 probably originated in the northeastern U.S., clinically emerging in hospital outbreaks as early as 2000. The subsequent success of ST258 has played a large role in the global dissemination of KPC through vertical transmission. We noted two instances where it appears ST258 replaced its Tn*4401* element through horizontal transmission. Why ST258 is closely linked to Tn*4401* is unknown. Given that ST258 is a healthcare-associated pathogen, a likely contributor to its selection is the heavy use of carbapenem antibiotics. In the late 1980s and early 1990s, clinicians relied on carbapenems as a last resort to battle the increasing number of Enterobacteriaceae producing extended-spectrum beta-lactamases (ESBLs). While carbapenem use continued during the time of ST258’s origination, a recent study by the Veteran’s Health Administration noted 102% increase in carbapenem use in their acute care facilities between 2005 and 2009 [63]. This increase was also noted in other U.S. hospitals, and mirrors the rapid expansion of ST258. The use of other antibiotics within the healthcare setting, particularly fluoroquinolones and aminoglycosides used to treat urinary tract infections (UTIs), could also act as a positive selective force, considering that ST258 causes UTIs and typically carries resistance mechanisms to several other classes of antibiotics.

The global spread pKpQIL-like plasmids, responsible for much of ST258’s Tn*4401* carriage [32–34] demonstrates the tenacity of particular plasmids. This plasmid analysis suggests the most recent common ancestor (MRCA) of ST258 Clades 1 and 2 carried a pKpQIL-like plasmid and it is highly persistent. It is impossible to determine whether pKpQIL-like plasmids date back further than the MRCA of Clades 1 and 2 from our data. It may have been in older ancestors and lost, or acquired multiple times. The vertical fidelity those plasmid types show in Clades 1 and 2 suggests it is not easily lost.

The antibiotic resistance gene patterns of CG258 do not illuminate a particular profile responsible for ST258 clonal success. CG258 isolates are similar, with the exception of the SNP variant genes’ *aac-Ib* and *aac-Ib-cr* mutual exclusivity. CG258 non-ST258 isolates have *aac-Ib-cr*, conferring fluoroquinolone resistance, while most ST258 have *aac-Ib*, conferring aminoglycoside resistance. Both groups carry at least one other aminoglycoside resistance gene and have the fluoroquinolone resistance mutations in *gyrA* and *parC*. Both aminoglycosides and fluoroquinolones are a highly used drug for UTIs, a common pathology of CG258 strains, and these multiple mechanisms for resistance to the same classes of antibiotics may offer higher resistance levels or resistance to different drugs within the same classes. Additionally, the amino acid change in the OqxAB repressor protein OqxR found in this study specific to CG258 isolates (Val130 to Ala) could suppress its repressor functions, resulting in overexpression of the OqxAB efflux system and high-level fluoroquinolone resistance. A similar mutation, Val102 to Gly, was responsible for a multidrug resistance phenotype in *K*. *pneumoniae* clinical isolates [50]. The deletion mutations that result in truncated OqxR proteins in three other clinical isolates studied here suggest a non-functional OqxR is not lethal and may offer a fitness advantage in certain circumstances. Further experiments are needed to test these hypotheses.

Two other potentially important mutations documented in this study, one in CG258 and the other specific to ST258, occur in a MarR family transcription repressor protein. MarR family proteins have been described in many species as responders to environmental stimuli such as host immune factors, toxins, antibiotics, and stress factors [41]. All CG258 isolates share a SNP in the *marR*-family gene resulting in amino acid change Arg4 to Ser. Although this change occurs outside of functionally characterized domains of the protein, it may affect protein structure and therefore function. The mutation specific to ST258 results from one of the four point mutations in the core genome separating ST258 from the rest of CG258. The resulting amino acid change, Ser34 to Phe, occurs in the homodimerization region of the protein. Given that this protein is highly conserved in the *K*. *pneumoniae* species, these amino acid changes may be significant. The Ser34 to Phe mutation may affect the ability to form a complete functional protein, bind stimuli ligands, or bind DNA to repress transcription. It is conceivable that suppression of the MarR family protein results in overexpression of systems that give ST258 a fitness advantage in particular environments. Further experiments to test this are underway. The SNP in ST258 that confers the amino acid change is now the target of a sensitive and specific assay to detect ST258. Additionally, another SNP in the *marR*-family gene encompassing all CG258 is the target of a second assay to detect CG258. SNPs are stable mutations, especially in highly conserved genes, and can be detected using a variety of molecular methods. Here we’ve shown that real-time PCR can be used for rapid detection and typing of *K*. *pneumoniae*. Both assays show 100% sensitivity and specificity, so are ideal for easy, cost-effective surveillance for ST258 and CG258.

Capsule modification allows adaptation to changing environments [53], and the variety of *cps* genotypes in our collection indicates that the capsule locus is highly mobile. IS elements reside within the *cps* region of some strains, potentiating the formation of new capsule types [18]. CG258 has at least 23 different capsule types, 11 uncharacterized, and ST258 at least three; one of which, firstly described in this study, lacks two highly conserved *cps* genes apparently deleted by integration of an IS element. Despite this disparity, the success of the strain does not appear to be affected. Some isolates had inconsistent genotypes in the capsule genes *wzc* and *wzi*: two shared a *wzc* but had different *wzi* genotypes, and some had identical genotypes but clearly different capsule types. These data, considered with the discovery of IS elements in several *cps* loci [18], should factor into interpretations of capsule typing by *wzc* and *wzi* sequencing. Several different capsule types characterize successful *K*. *pneumoniae* clinical pathogens, and as more isolates are sequenced, more and more types will undoubtedly be found. The limited number of capsule types characterizing ST258 make the capsule a good target for a vaccine, however surveillance will be critical to detect any future recombination events.

The functional repertoire of outer membrane proteins in *K*. *pneumoniae* is vast and complex, but undoubtedly includes functions critical to environmental adaptation. Depending on the allele, OmpA may cause more or less invasive capacity, immune evasion, adherence to particular cells or surfaces, and can affect frequency of plasmid conjugal receipt from donors [56]. The conservation in OmpA sequence in our collection may reflect selection against mutation. Likewise, OmpK26 was conserved despite the isolate diversity, suggesting selection against mutation. OmpK26 compensates for dual OmpK35 and OmpK36 loss in clinical isolates [57], and may play a role in compensating for OmpK35 loss alone, which we found is common in this collection of isolates. Conversely, we found 40 different OmpK36 sequences in our isolates, with amino acid variation concentrated in the extracellular loop regions. Selection for variation in OmpK36 may slow host recognition, or allow colonization of new tissues, as these are functions in the OmpK36 repertoire [55]. Outer membrane protein analysis revealed a profile in all ST258 that includes a truncated OmpK35, which would be expected to have deleterious effects on fitness, but may provide a degree of positive selection in a host environment where OmpK35 is not typically expressed [64]. In our analyses, protein truncation was much more frequent in OmpK35 than in other outer membrane proteins. The OmpK37 sequence found in our ST258 and CG258 isolates contains insertions in the extracellular loop regions, which again may impact interaction with its environment. ST258’s OMP profile, including OmpK35 loss and OmpK37 extended loops, could contribute to its enhanced ability to persist in a host or healthcare environment.

Our data underscore the usefulness of whole genome sequencing in epidemiology, evolutionary history, and specific genetic attributes of pathogens. The genomic analyses of KPC-producing *K*. *pneumoniae* that we present in this study provide further insight into the evolution and rapid spread of the globally dominant strain, ST258. We show that in addition to the large recombination events that gave rise to CG258 and ST258 [17, 20], key point mutations may also play a significant role in the evolution of these strains. Based on these SNPs, the limited number of *cps* variations and the OMP profile that is conserved within ST258, this work also provides information important to surveillance and to development of a vaccine to specifically target ST258 and contain the KPC-producing *K*. *pneumoniae* pandemic.

## Materials and Methods

### Strain collection

This study’s *K*. *pneumoniae* isolated in the United States (n = 72) were selected from the CDC’s collection, which primarily comprises isolates submitted for reference testing or as part of an outbreak investigation in which the CDC was involved. Selection criteria were based on PFGE profiles, MLST sequence types when available, geography, year of isolation, and KPC status. U.S. isolates were selected with a focus on ST258, followed by other CG258 and non-CG258 isolates. Isolates from other countries (n = 95) were generously donated upon a request to various countries with recent reports of KPC-producing ST258 or CG258 strains (see Acknowledgments). S1 and S2 Tables describe the isolate collection.

### Sequencing, MLST, and SNP detection

Genome libraries were prepared with a 500 base pair insert size using a KAPA Library Preparation Kit with Standard PCR Library Amplification (Kapa Biosystems, Wilmington, MA) and sequenced on a 101 bp read, paired-end Illumina GAIIx run. SRST2 [65] was used to determine multilocus sequence types. NASP, a pipeline developed by TGen North (https://github.com/TGenNorth/NASP), was used to detect SNPs. In brief, reads were aligned to the finished *K*. *pneumoniae* genomes MGH 78578 (GenBank accession no. CP000647) or the ST258 reference genome NJST258_1 (GenBank CP006923) using Novoalign (Novocraft.com) and SNPs called with GATK [66]. Data filtered out included SNP loci with less than 10X coverage or with less than 90% consensus in any one sample, regions duplicated in the reference genome as identified by Nucmer, and SNP loci that were not present in all genomes in the dataset. The results were output in a SNP matrix from a core genome common to all isolates in the analysis. Core genome size is expressed as the size of the reference genome (or percentage of the total reference genome size) excluding repeated regions and covered by reads at 10X or higher depth by all samples, or the length of the DNA that all samples in a given set have in common after filtering based on the above criteria. Read data were deposited in the NCBI SRA database under BioProject ID PRJNA252957.

### Phylogenetic analysis

Phylogenetic trees were generated from the SNP matrices using the maximum parsimony method with 1000 bootstraps in MEGA 5.2 [67] [68]and subsequently plotted by means of ITOL v2 [69]. The genome of a *K*. *oxytoca* isolate (GenBank accession no. CP003218) was used as the outgroup to root an initial *K*. *pneumoniae* tree. The isolates with the basal-most branch, or the isolates with the branch closest to the outgroup, was used as the outgroup to root the following tree without *K*. *oxytoca*. All subsequent trees to analyze a progressively smaller number of isolates used the isolates with the basal-most branch from the previous tree as the root.

Bayesian evolutionary analysis was performed in BEAST v1.7.4 [70] using the SNP matrix generated by NASP to compute evolutionary rates and divergence times using the GTR model of nucleotide substitution and an uncorrelated lognormal relaxed clock. A tree prior of exponential growth was used along with a random starting tree and an exponential growth rate set to random walk. Isolates were dated based on the year of isolation and were run with 50 million generations and a burn-in phase of 5 million. Three independent Markov Chain Monte Carlo analyses were completed and combined in order for all parameters’ effective sample size values to be larger than 500.

### Targeted genome analysis

Plasmid incompatibility groups were detected *in silico* by uploading read data to PlasmidFinder [71]. Known horizontally transferred antibiotic resistance genes were detected with SRST2 [65, 72]. Selected genes were also aligned to a reference gene with SeqMan NGen (DNASTAR, Madison, WI) to confirm their presence and type in read data. Reads were assembled using SPAdes Genome Assembler [73] after trimming Illumina adaptors with Trimmomatic [74]. Porin sequences were analyzed using SSTAR (https://github.com/tomdeman-bio/Sequence-Search-Tool-for-Antimicrobial-Resistance-SSTAR-) and Geneious [75], and *cps* loci and Tn*4401* were characterized using Geneious [75] and SeqMan NGen (DNASTAR, Madison, WI). Capsule types were assigned using the *wzc* and *wzi* sequence databases in BIGSdb ([76, 77], http://bigsdb.web.pasteur.fr/klebsiella/).

### SNP assays

Real-time PCR assays targeting the SNPs specific to ST258 and CG258 were designed with Biosearch Technologies’ RealTimeDesign software (Biosearch Technologies, Petaluma, CA). Assays were run in 10uL reactions on the 7900HT instrument (Life Technologies, Carlsbad, CA) with 1X PerfeCTa qPCR FastMix II (Quanta Biosciences, Gaithersburg, MD), 600 nM forward and reverse primers, 200 nM each probe, and 1 μL DNA template (approximately 0.5ng). Thermal conditions included denaturation for 4 min at 95°C followed by 40 cycles of 15 s at 95°C and 1 min at 60°C.

## Supporting Information

S1 Fig A maximum parsimony phylogeny based on 1,736 core genome SNPs in 208 ST258 isolates using the ST258 reference genome NJST258_1 (GenBank accession no. CP006923) with the 215 kb region of recombination [15] masked. 101 isolates are from this study, 83 are from DeLeo *et al*. [15] and were retrieved from the SRA database of NCBI (Study no. SRP036874,), 22 are from the outbreak at the National Institutes of Health described by Snitkin *et al*. [23] and were retrieved as assemblies from Genbank. The US-GA isolates that were in Clade 2 previously but fall outside Clade 2 in this phylogeny are in the dotted box. Consistency index = 0.97.
(PDF)

S2 Fig Alignment of MarR family proteins. Figure recreated from Wilkinson and Grove [78], with the addition of the MarR family amino acid sequence described in this study (bottom sequence). The amino acid substitution specific to ST258 in the α1 region is boxed. Light and dark shading indicates >70% similarity or >70% identity at that position respectively. α = alpha helices, β = beta turns, W = wing. The helix-turn-helix motif corresponds to helices α3 and α4, and helices α1, α5, and α6 form the dimerization domain [78].
(PDF)

S1 Table Geographic and temporal diversity in our collection of *K*. *pneumoniae*. (DOCX)

S2 Table List of study isolates and associated data. (XLSX)

S3 Table Sequence assembly statistics. (XLSX)
