# Supplementary figures and images for: Genomic Analysis of the Emergence and Rapid Global Dissemination of the Clonal Group 258 Klebsiella pneumoniae Pandemic
Source: PLoS One. 2015 Jul 21;10(7):e0133727. doi: 10.1371/journal.pone.0133727 (PMC4510304; doi:10.1371/journal.pone.0133727)

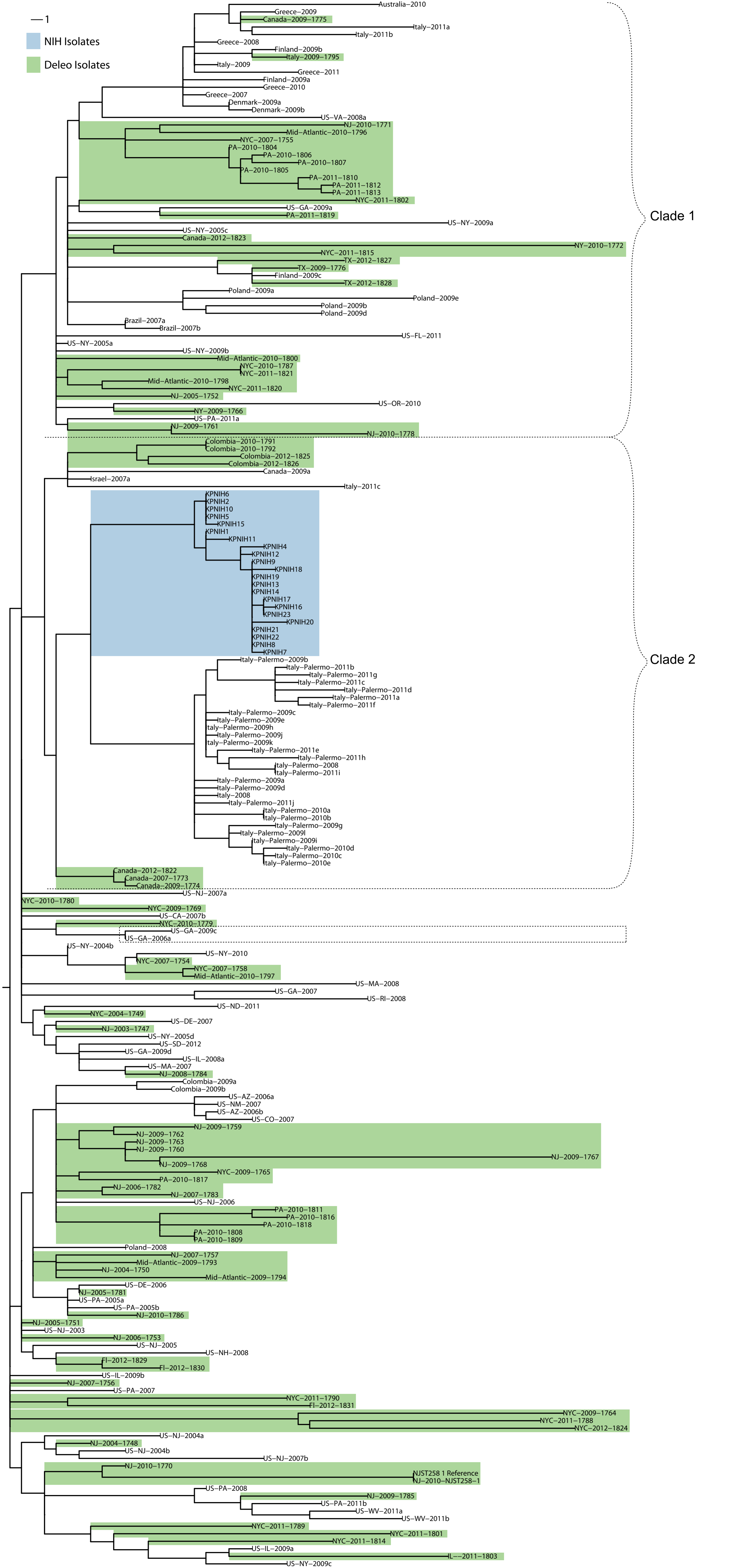

Supplement: S1 Fig — 101 isolates are from this study, 83 are from DeLeo et al. [15] and were retrieved from the SRA database of NCBI (Study no. SRP036874,), 22 are from the outbreak at the National Institutes of Health described by Snitkin et al. [23] and were retrieved as assemblies from Genbank. The US-GA isolates that were in Clade 2 previously but fall outside Clade 2 in this phylogeny are in the dotted box. Consistency index = 0.97. (PDF) [file pone.0133727.s001.pdf]

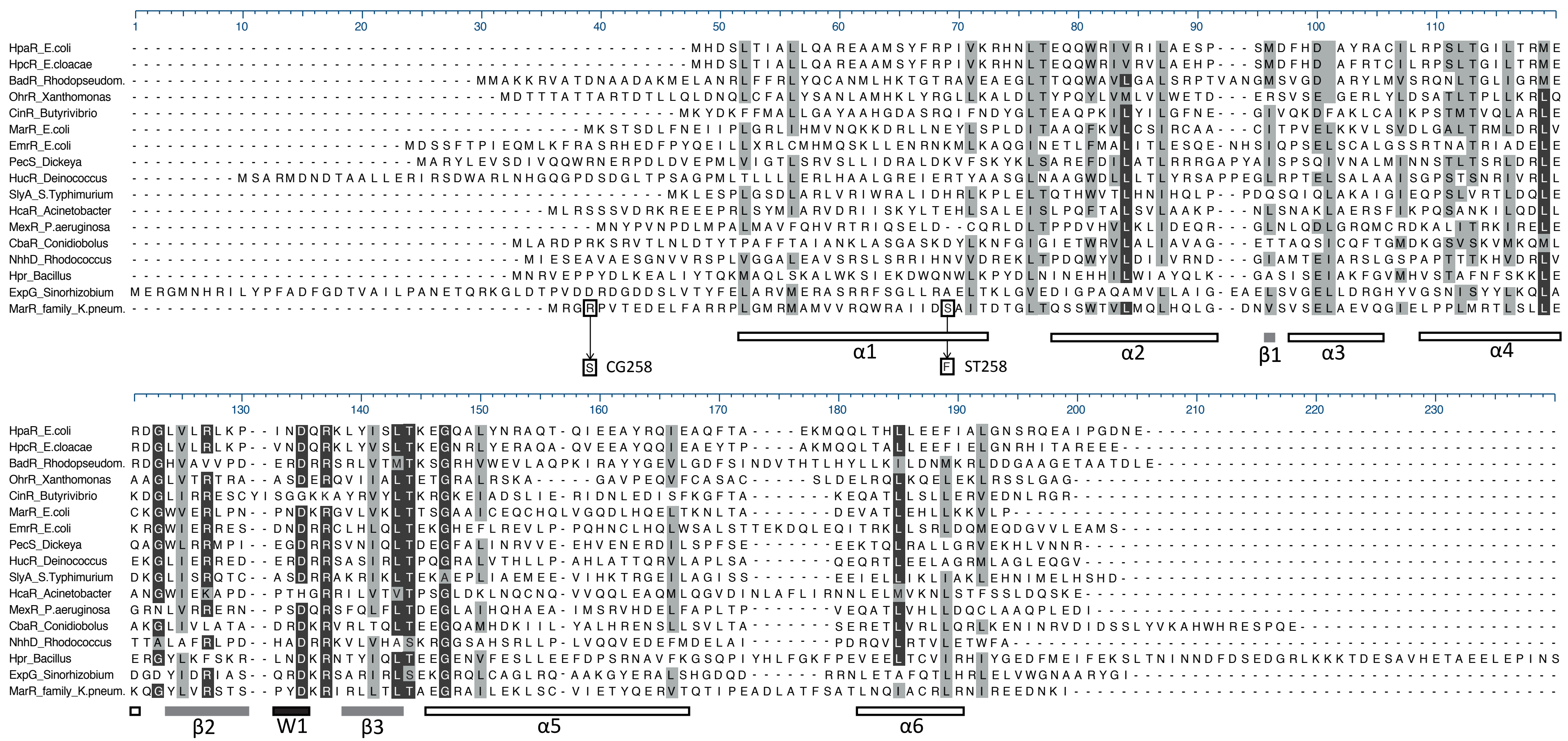

Supplement: S2 Fig — Figure recreated from Wilkinson and Grove [78], with the addition of the MarR family amino acid sequence described in this study (bottom sequence). The amino acid substitution specific to ST258 in the α1 region is boxed. Light and dark shading indicates >70% similarity or >70% identity at that position respectively. α = alpha helices, β = beta turns, W = wing. The helix-turn-helix motif corresponds to helices α3 and α4, and helices α1, α5, and α6 form the dimerization domain [78]. (PDF) [file pone.0133727.s002.pdf]
