# Supplementary material for: Genomic Analysis of the Emergence and Rapid Global Dissemination of the Clonal Group 258 Klebsiella pneumoniae Pandemic
Source: PLoS One. 2015 Jul 21;10(7):e0133727. doi: 10.1371/journal.pone.0133727 (PMC4510304; doi:10.1371/journal.pone.0133727)
Supplement: S1 Table — (DOCX) [file pone.0133727.s003.docx]

| Global Collection | | | | | | | | | United States Collection | | | | | |
| --- | --- | --- | --- | --- | --- | --- | --- | --- | --- | --- | --- | --- | --- | --- |
| **Country** | Isolates | CC11 | | ST258 | | Unrelated | | Year of Isolation | State | Isolates | CC11 | ST258 | Unrelated | Year of Isolation |
| **Australia** | 1 | 1 | | 0 | | 0 | | 2010 | **AZ** | 2 | 2 | 2 | 0 | 2006 |
| **Brazil** | 12 | 12 | | 2 | | 0 | | 2006 - 2010 | **CA** | 6 | 4 | 1 | 2 | 2007 - 2011 |
| **Canada** | 4 | 1 | | 0 | | 3 | | 2009 | **CO** | 1 | 1 | 1 | 0 | 2007 |
| **Colombia** | 2 | 2 | | 2 | | 0 | | 2009 | **DE** | 2 | 2 | 2 | 0 | 2006 - 2007 |
| **Denmark** | 2 | 2 | | 2 | | 0 | | 2009 | **FL** | 2 | 1 | 1 | 1 | 2008 - 2011 |
| **Finland** | 3 | 3 | | 3 | | 0 | | 2009 | **GA** | 7 | 5 | 5 | 2 | 2006 - 2009 |
| **Greece** | 5 | 5 | | 5 | | 0 | | 2007 - 2011 | **IL** | 4 | 3 | 3 | 1 | 2008 - 2009 |
| **Guatemala** | 1 | 1 | | 0 | | 0 | | 2009 | **MA** | 2 | 2 | 2 | 0 | 2007 - 2008 |
| **Hong Kong** | 1 | 1 | | 0 | | 0 | | 2008 | **MD** | 3 | 0 | 0 | 3 | 2005 - 2011 |
| **India** | 5 | 3 | | 0 | | 2 | | 2007 - 2008 | **MI** | 1 | 0 | 0 | 1 | 2008 |
| **Indonesia** | 1 | 1 | | 0 | | 0 | | 2008 | **MO** | 1 | 0 | 0 | 1 | 2006 |
| **Israel** | 4 | 2 | | 0 | | 2 | | 2007 | **NC** | 1 | 0 | 0 | 1 | 1996 |
| **Italy** | 33 | 32 | | 31 | | 1 | | 2008 - 2011 | **ND** | 1 | 1 | 1 | 0 | 2011 |
| **Korea** | 1 | 1 | | 0 | | 0 | | 2008 | **NH** | 1 | 1 | 1 | 0 | 2008 |
| **Malaysia** | 2 | 2 | | 0 | | 0 | | 2008 - 2009 | **NJ** | 7 | 7 | 7 | 0 | 2003 - 2007 |
| **Philippines** | 1 | 1 | | 0 | | 0 | | 2009 | **NM** | 1 | 1 | 1 | 0 | 2007 |
| **Poland** | 6 | 6 | | 5 | | 0 | | 2008 - 2009 | **NY** | 12 | 8 | 8 | 4 | 2004 - 2010 |
| **Singapore** | 1 | 1 | | 0 | | 0 | | 2008 | **OR** | 1 | 1 | 1 | 0 | 2010 |
| **Thailand** | 10 | 10 | | 0 | | 0 | | 2007 - 2008 | **PA** | 7 | 6 | 6 | 1 | 2001 - 2011 |
| **United States** | 70 | 50 | | 47 | | 20 | | 1996 - 2012 | **RI** | 1 | 1 | 1 | 0 | 2008 |
|  |  |  | |  | |  | |  | **SD** | 1 | 1 | 1 | 0 | 2012 |
| **Total** | **167** | **137** | | **97** | | **28** | |  | **TX** | 2 | 0 | 0 | 2 | 2001 - 2011 |
|  |  |  | |  | |  | |  | **VA** | 3 | 1 | 1 | 2 | 2007 - 2008 |
|  |  | | | | | | |  | **WA** | 1 | 0 | 0 | 1 | 2010 |
|  |  | |  | |  | |  |  | **WV** | 2 | 2 | 2 | 0 | 2011 |
